# Essential phospholipids impact cytokine secretion and alter lipid-metabolizing enzymes in human hepatocyte cell lines

**DOI:** 10.1007/s43440-024-00595-4

**Published:** 2024-04-26

**Authors:** Dominik Wupperfeld, Gert Fricker, Béatrice Bois De Fer, Branko Popovic

**Affiliations:** 1 Department of Pharmaceutical Technology and Biopharmacy, Institute of Pharmacy and Molecular Biotechnology, Ruprecht-Karls University of Heidelberg, Heidelberg, Germany; 2 Sanofi, Neuilly-Sur-Seine, France; 3 Sanofi, Frankfurt am Main, K607, 65929 Industriepark Hoechst Germany

**Keywords:** Essential phospholipids, HepG2, HepaRG, Non-alcoholic fatty liver disease, Steatotic HepaRG

## Abstract

**Background:**

Essential phospholipids (EPL) are hepatoprotective.

**Methods:**

The effects on interleukin (IL)-6 and -8 secretion and on certain lipid-metabolizing enzymes of non-cytotoxic concentrations of EPL (0.1 and 0.25 mg/ml), polyenylphosphatidylcholine (PPC), and phosphatidylinositol (PtdIns) (both at 0.1 and 1 mg/ml), compared with untreated controls, were assessed in human hepatocyte cell lines (HepG2, HepaRG, and steatotic HepaRG).

**Results:**

Lipopolysaccharide (LPS)-induced IL-6 secretion was significantly decreased in HepaRG cells by most phospholipids, and significantly increased in steatotic HepaRG cells with at least one concentration of EPL and PtdIns. LPS-induced IL-8 secretion was significantly increased in HepaRG and steatotic HepaRG cells with all phospholipids. All phospholipids significantly decreased amounts of fatty acid synthase in steatotic HepaRG cells and the amounts of acyl-CoA oxidase in HepaRG cells. Amounts of lecithin cholesterol acyltransferase were significantly decreased in HepG2 and HepaRG cells by most phospholipids, and significantly increased with 0.1 mg/ml PPC (HepaRG cells) and 1 mg/ml PtdIns (steatotic HepaRG cells). Glucose-6-phosphate dehydrogenase activity was unaffected by any phospholipid in any cell line.

**Conclusions:**

EPL, PPC, and PtdIns impacted the secretion of pro-inflammatory cytokines and affected amounts of several key lipid-metabolizing enzymes in human hepatocyte cell lines. Such changes may help liver function improvement, and provide further insights into the EPL’s mechanism of action.

**Supplementary Information:**

The online version contains supplementary material available at 10.1007/s43440-024-00595-4.

## Introduction

Non-alcoholic fatty liver disease (NAFLD) is one of the most common chronic liver diseases [1]. Disease progression includes simple fatty liver, non-alcoholic steatohepatitis (NASH), fatty liver cirrhosis, and hepatocellular carcinoma [1, 2]. No FDA-approved drugs are available for treating NAFLD/NASH. Current treatments focus on lifestyle changes (e.g., weight loss, exercise; [3–5]). Certain medications such as herbal preparations [6] and essential phospholipids (EPL) [7, 8] are adjunctive treatments for NAFLD/NASH [9].

Essentiale® is an EPL preparation consisting of several phospholipids; mainly polyenylphosphatidylcholine (PPC) and phosphatidylinositol (PtdIns) [10]. Phospholipids are key elements in mammalian cells, e.g., phosphatidylcholines (PtdChos) are involved in several cellular functions [11], and phosphoinositides in cell polarity [12]. The hepatoprotective effects of EPL are widely studied [7, 8, 11, 13, 14]. Moreover, clinical benefits of EPL have been demonstrated in NAFLD [7], viral hepatitis [11], and in the treatment/prevention of chemical- and drug-induced toxicity [11]. In alcoholic liver disease (ALD), EPL have been found to protect against fibrosis in animal models including baboons [15] and mice [16], with a putative mechanism of action involving the downregulation of cytochrome P450 2E1 (CYP2E1) and reduction of CYP2E1-derived oxidative stress, as well as reduction in acetyl-CoA oxidase (ACOX1) expression [16].

Hepatocytes have multiple functions [17], any of which are potential targets for EPL. Several studies have demonstrated multiple properties of EPL, including effects on membrane-dependent cellular functions as well as anti-apoptotic, antifibrogenic, anti-inflammatory, antioxidant, antisteatotic, lipid-regulating, and membrane protective effects [11, 13, 14]. However, data is lacking on these effects of EPL in human hepatocytes.

Lipopolysaccharides (LPS), which are the major component of the outer surface of Gram-negative bacteria, stimulate the secretion of numerous pro-inflammatory cytokines from a wide variety of cells [18] and are key cofactors in liver injury [19]. The mechanisms via which LPS cause liver injury are not fully elucidated, however there is recent evidence to suggest that ferroptosis resulting from lipid peroxidation caused by reactive oxygen species (ROS) may play a major role [20, 21].

Recent investigations focused on the effects of EPL, PPC, and PtdIns in several human hepatocyte cell lines (HepG2, HepaRG, and steatotic HepaRG) on membrane fluidity, apoptosis, and hepatocyte transporter function [22]. All three increased membrane fluidity in HepG2 cultures, as did PtdIns in steatotic HepaRG cells. Tamoxifen-induced apoptosis in HepG2 cells was significantly decreased by EPL, PPC, and PtdIns. Several hepatocellular export proteins were also impacted in vitro, i.e., increased breast cancer resistance protein activity (by EPL and PtdIns in HepG2 cells); increased multidrug resistance-associated protein 2 activity (by all three phospholipids in HepaRG cells, and by PtdIns in steatotic HepaRG cells); increased bile salt export protein activity (by EPL and PPC in HepG2 and steatotic HepaRG cells); increased P-glycoprotein activity (by all phospholipids in HepG2 cells, and by PtdIns HepaRG and steatotic HepaRG cells) [22]. Thus, these in-vitro investigations provided valuable insights into human hepatocytes of the mechanism of action of EPL. Two other biochemical pathways involved in NAFLD pathogenesis (inflammation, lipid dysregulation) and impacted by EPL in vivo are also amenable to further in-vitro investigations in human hepatocytes i.e., pro-inflammatory cytokines and lipid-metabolizing enzymes.

Cytokines are critical in health/disease [23]. Pro-inflammatory cytokines (e.g., interleukins [ILs]) are involved in NAFLD pathogenesis as they stimulate hepatic inflammation, steatosis, cell apoptosis and necrosis, and induce fibrosis [24]. A meta-analysis of 51 studies of 19 different pro-inflammatory cytokines, involving >36,000 patients with NAFLD and >47,000 healthy controls, showed that increased levels of C-reactive protein, IL-1β, IL-6, tumor necrosis factor-α and intercellular adhesion molecule 1 were significantly associated with NAFLD [25]. The IL-6 family of cytokines is essential in metabolism. Genetically engineered mouse models showed numerous effects of IL-6 cytokines in metabolic homeostasis, including NASH [26]. Interestingly, blocking IL-6 signaling with anti-IL-6Rα enhanced steatosis but reduced inflammation, thereby supporting the role of IL-6 trans-signaling in inflammation [26]. Previous reports showed that hepatic IL-6 signaling had a protective role against hepatic steatosis progression while enhancing liver inflammation in mice [27]. In patients with chronic liver disease, IL-8 serum levels were significantly higher versus healthy controls, particularly in those with end-stage cirrhosis [28]. Furthermore, IL-8 levels correlate with liver function and non-invasive fibrosis and likely contribute to hepatic inflammation [28]. Thus, IL-6 and IL-8 are valid endpoints to evaluate in human hepatocyte cell lines to examine the mechanism of action of EPL and its components.

The key pathophysiological feature of NAFLD is abnormal lipid accumulation [29], and the liver has a complex role in lipid homeostasis [30]. Key enzymes involved in lipid metabolism, easily measurable in vitro, include FAS, ACOX1, LCAT, and glucose-6-phosphate dehydrogenase (G6PD).

FAS catalyzes saturated fatty acid biosynthesis [31]. Incubation of primary human hepatocytes in vitro with fatty acids induced lipid accumulation and FAS expression [32]. In mice with hepatic steatosis, hepatic FAS expression was increased versus controls [32]. ACOX1 is a rate-limiting step in peroxisomal fatty acid oxidation [33]. Studies in mice with mutated ACOX1 had accelerated NAFLD progression [34]. ACOX1 expression in liver samples from patients with NAFLD was double that in healthy people [35]. LCAT is a key enzyme in high-density lipoprotein metabolism [36]. Patients with NAFLD displayed high plasma LCAT activity [37]. G6PD activity in the pentose phosphate pathway produces NADPH; a vital component in fatty acid biosynthesis [38]. Increased G6PD is associated with lipid metabolism dysregulation [38] and fatty liver in rats [39]. Therefore, evaluating these enzymes in human hepatocyte cell lines is valid in understanding the EPL mechanism of action.

Although the ultimate cells for in-vitro evaluations of liver cellular mechanisms are primary human hepatocytes, these are not readily available and necessitate using immortal hepatic cell lines [40]. Common human hepatocyte cell lines include HepG2 and HepaRG cells [41, 42]. HepaRG cells can be converted to steatotic cells as a model system for evaluating fatty liver disease [43, 44].

This study aimed to evaluate the effects of EPL, PPC, and PtdIns on the secretion of certain pro-inflammatory cytokines, and the effects of these phospholipids on key enzymes involved in lipid metabolism in HepG2, HepaRG and steatotic HepaRG cell lines. These evaluations extend previously reported findings on the EPL mechanism of action as a hepatoprotectant in NAFLD [22].

## Materials and methods

### Preparation of liposomes

As aqueous cell culture media were used, all phospholipid preparations were incorporated into liposomes so that sufficiently high concentrations were achieved in vitro. The methods have been described previously [22]; see supplementary information.

### Cell lines and culture conditions

Cell lines and culture conditions have been described previously [22]. HepG2 cells were seeded at a 1.4 × 10^5^ cells/cm², and cultured in Roswell Park Memorial Institute 1640 medium supplemented with 10% FBS, 100 U/ml penicillin, and 100 µg/ml streptomycin. Fully differentiated HepaRG cells were cultured as described previously by Le Guillou et al. [45]. HepaRG cells were seeded at 2.25 × 10^5^ cells/cm² in Williams’ medium E supplemented with 5% FBS, 100 U/ml penicillin, 100 µg/ml streptomycin, 2 mM glutamine, 5 µg/ml insulin, 50 µM hydrocortisone hemisuccinate, and 1% DMSO. Steatosis was induced by treating HepaRG cells for 2 weeks with 150 µM stearic acid and 150 µM oleic acid, which were dissolved in DMSO (final DMSO concentration was 1%). All cell lines were cultured at 37^o^C (atmosphere:5% CO_2_; 95% humidity), with renewal of culture medium every 2 or 3 days.

### Effect of phospholipids on secretion of pro-inflammatory cytokines interleukin (IL)-6 and IL-8

LPS was the positive control for cytokine release. To select the most appropriate LPS concentration and incubation times, HepaRG cells were incubated with LPS (0–2,500 ng/ml) for 2–24 h, and IL-8 levels were determined in the cell supernatant (see below).

Seeding and culture of HepaRG and steatotic HepaRG cells was as previously described. Phospholipids were added to these cell lines as liposomal preparations (see above) at concentrations identified previously [22] i.e., EPL at 0.1 and 0.25 mg/ml; and PPC and PtdIns at 0.1 and 1.0 mg/ml. These concentrations were considered the highest concentrations that could be used in hepatocyte cultures without inducing major cytotoxicity [22]. The cytotoxicity of these phospholipids was evaluated in previous experiments in HepG2 and HepaRG cell lines using a cell viability reagent [22]. In HepG2 cell line cultures, EPL concentrations of ≥0.375 mg/ml were markedly cytotoxic, PPC concentrations of >0.9 mg/ml were moderately cytotoxic, and PI at concentrations 0.36 to 7.3 mg/ml resulted in modest decreases in cell viability cytotoxic [22]. None of the evaluated concentrations of these three phospholipids (0.01 to 20 mg/ml) demonstrated cytotoxicity in the HepaRG cell line in culture [22]. In these experiments, phospholipid-treated hepatocyte cell lines were incubated for 48 h.

To evaluate IL-6 and IL-8 secretion, phospholipid-treated cells were treated with 1.5 µg/mL LPS and incubated at 37^o^C for 24 h. Cell culture supernatants were collected and stored at − 80^o^C until assayed. IL-6 and IL-8 were quantified using ELISA (QIAGEN Cat. #: SEH00560A and SEH00568A, respectively) according to the manufacturer’s instructions. Absorption was measured using a Tecan Sunrise Vis-photometry plate reader (450 nm wavelength). IL-6 and IL-8 concentrations were determined using standard curves.

### Evaluation of lipid-metabolizing enzymes

The three cell lines were cultured as described above. HepG2 cells were seeded in T-75 flasks to achieve sufficient amounts of the enzymes to be investigated. HepaRG cells were seeded in 12-well culture plates, and steatotic HepaRG cells were seeded in 6-well culture plates at 2.25 × 10^5^ cells/cm². Phospholipids were added to these cell lines as liposomal preparations (see above) i.e., EPL at 0.1 and 0.25 mg/ml; PPC and PtdIns at 0.1 and 1.0 mg/ml. Phospholipid-treated hepatocyte cell lines were incubated for 48 h.

Cells were washed twice with warm PBS, and trypsinized for 10 min; this process was stopped by the addition of the appropriate cell culture medium. The trypsinized cells were then centrifuged at 500 xg for 5 min and the supernatant was discarded. The cell pellet was resuspended in 1 ml PBS (HepG2 cells) or in 250 µl PBS (HepaRG and steatotic HepaRG cells) and stored frozen at − 20 °C. To lyse the cells, frozen cells were thawed once and frozen at − 80 °C until analysis. Prior to conducting the enzyme assays, lysed cells were thawed and centrifuged at 15,000 xg for 10 min at 4 °C. The supernatant was used for the different enzyme assays (see the supplementary information for further details).

### Statistical analyses

The sample size was not determined formally. SAS^®^ software version 9.4 or higher (SAS Institute Inc. Cary NC USA) was used for the data analyses. Data were displayed graphically, and each parameter was analyzed descriptively (n, arithmetic mean, and standard deviation).

Each experiment was conducted four to five times, and within each experiment one replicate per treatment was performed. Values from each experiment were then used to calculate means and standard errors for each parameter. For each cell line and parameter, comparisons were made between each treatment group (EPL, PPC, PtdIns at different concentrations) versus untreated controls (non-phospholipid treated cells). For IL-6 and IL-8 concentrations, the amounts of FAS, ACOX1, and LCAT, and G6PD activity, comparisons were made using analysis of variance (ANOVA; least square [LS] means, standard error, 95% confidence interval [CI]) including treatment group as a fixed factor and Dunnett’s adjustment for pairwise comparisons. For untreated cells only, comparisons across cell lines were performed for each parameter using an ANOVA including cell line as a fixed factor and Dunnett’s adjustment, with the steatotic HepaRG cell line considered as the control group. A threshold of 5% was used to denote statistical significance for all pairwise comparisons.

Further details regarding reagents and equipment are listed in the Supplementary section.

## Results

### Pro-inflammatory cytokine secretion in human hepatocyte cell lines

Twelve pro-inflammatory cytokines (IL-1 A, IL-1B, IL-2, IL-4, IL-5, IL-6, IL-8, IL-10, IL-12, IFNγ, TNFα, TGFb1) were assessed in HepG2 and HepaRG cell lines. Based on the concentration-response and time course of LPS induction of IL-8 secretion (supplemental Fig. S1), 1.5 µg/mL LPS for 24 h was selected for inducing the highest secretion of cytokines.

In HepG2 cells, TNFα, TGFb1, and IL-8 were not induced by LPS; the remaining nine were undetectable. Thus, experiments were only performed with the two HepaRG cell lines. However, in both HepaRG and steatotic HepaRG cell lines, the measured signals of IL-1 A, IL-1B, IL-2, IL-4, IL-5, IL-10, IL-12, IFNγ, TNFα, and TGFb1 in the cell medium as well as in cell lysates were very low, precluding reliable results. Furthermore, these were not induced by LPS (data not shown).

IL-6 and IL-8 were the only measurable and LPS-inducible pro-inflammatory cytokines in HepaRG and steatotic HepaRG cell lines; thus further work focused on these two.

### Impact of phospholipids on IL-6 secretion

In untreated cells (no LPS and no phospholipid addition), IL-6 secretion by HepaRG cells was significantly lower than steatotic HepaRG cells; LS mean difference (95% CI) was − 43.2 (–67.3 to − 19.2), *p* < 0.01.

In HepaRG cells with no LPS addition, IL-6 secretion was significantly decreased by EPL at 0.1 mg/ml (LS mean difference [95% CI] − 4.3 [–8.3 to − 0.2] pg/ml; *p* < 0.05), and significantly increased with 1 mg/ml PtdIns (8.7 [4.7 to 12.8] pg/ml; *p* < 0.001) compared with untreated cells. All other observations with phospholipid addition to HepaRG cells (no LPS) were not statistically significant versus untreated cells (data not shown).

In steatotic HepaRG cells (no LPS addition), IL-6 secretion was significantly decreased by both concentrations of EPL (LS mean difference [95% CI] 0.1 mg/ml: − 27.9 [–46.7 to − 9.2] pg/ml, *p* < 0.01; 0.25 mg/ml: − 23.2 [–42.0 to − 4.4] pg/ml, *p* < 0.05) versus untreated cells. IL-6 secretion was also significantly decreased by only the low concentrations of PPC (0.1 mg/ml: − 20.0 [–38.8 to − 1.2] pg/ml, *p* < 0.05) and PtdIns (0.1 mg/ml: − 21.1 [–39.8 to − 2.3], *p* < 0.05) compared with untreated cells.

**Fig. 1:**
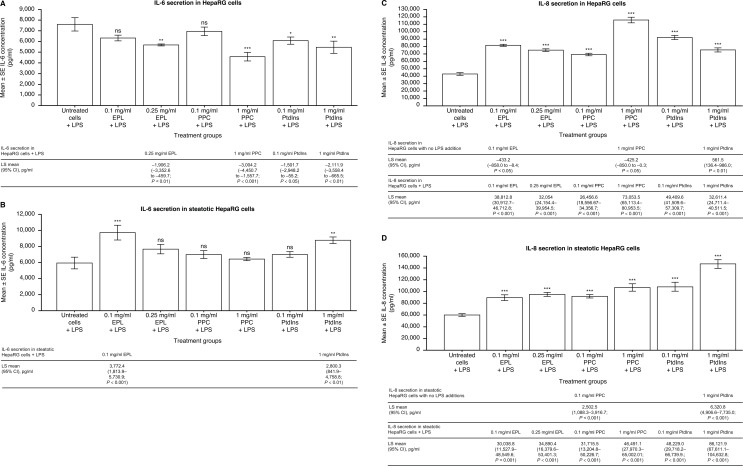
Effect of EPL, PPC, and PtdIns on LPS-induced secretion of IL-6 and IL-8 in the HepaRG and steatotic HepaRG cell lines. Values shown are LS mean ± SE for five separate experiments; *n* = 1 well for each concentration of each compound per experiment. *p < 0.05; **p < 0.01; ***p < 0.001 versus untreated cells (ANOVA with Dunnett adjustment for pairwise comparisons). ANOVA, analysis of variance; CI, confidence interval; EPL, essential phospholipid; IL, interleukin; LPS, lipopolysaccharides; LS, leastsquares; ns, not significant; PtdIns, phosphatidylinositol; PPC, polyenylphosphatidylcholine; SE, standard error

The addition of LPS to HepaRG cells, in the absence of phospholipids, increased IL-6 secretion compared with cells without LPS i.e., LS means (95% CI) were 7,605.7 (6,838.7–8,372.8) pg/ml versus 18.2 (16.1–20.4) pg/ml. Statistically significant decreases in LPS-induced IL-6 secretion were seen with all phospholipids evaluated compared with LPS-treated HepaRG cells (Fig. 1A). Both concentrations of PtdIns significantly decreased LPS-induced IL-6 secretion versus non-phospholipid treated HepaRG cells.

The addition of LPS to steatotic HepaRG cells, in the absence of phospholipids, increased IL-6 secretion compared with cells without LPS i.e., LS means (95% CI) were 5,941.6 (4,903.0–6,980.2) pg/ml versus 61.5 (51.5–71.4) pg/ml. Statistically significant increases in LPS-induced IL-6 secretion were seen with EPL and PtdIns addition compared with LPS-treated steatotic HepaRG cells (Fig. 1B).

### Impact of phospholipids on IL-8 secretion

In untreated cells (no LPS and no phospholipid addition), IL-8 secretion by HepaRG cells was significantly lower than by steatotic HepaRG cells; LS mean difference (95% CI) was − 762.9 (–1,442.7 to − 83.1) pg/ml, *p* < 0.05.

In HepaRG cells with no LPS addition, IL-8 secretion was significantly decreased by EPL and PPC addition. PtdIns addition at 1 mg/ml to HepaRG cells in the absence of LPS significantly increased IL-8 secretion. All other observations on IL-8 secretion with phospholipid addition to HepaRG cells (no LPS) were not statistically significant versus untreated cells (data not shown).

In steatotic HepaRG cells with no LPS addition, IL-8 secretion was significantly increased by PPC and PtdIns addition versus untreated cells. All other observations on IL-8 secretion with phospholipid addition to steatotic HepaRG cells (no LPS) were not statistically significant versus untreated cells (data not shown).

Addition of LPS to HepaRG cells, in the absence of phospholipids, a higher amount of IL-8 secretion was observed versus with cells without LPS i.e., LS means (95% CI) were 42,553.8 (38,364.3, 46,743.3) pg/ml versus 1,429.7 [1,204.4, 1,655.0] pg/ml), respectively.

Statistically significant increases in LPS-induced IL-8 secretion were seen with all phospholipids at all concentrations evaluated compared with LPS-treated HepaRG cells (Fig. 1C).

Statistically significant increases in LPS-induced IL-8 secretion were seen with all phospholipids at all concentrations evaluated compared with LPS-treated steatotic HepaRG cells (Fig. 1D).

### Impact of phospholipids on the amount of FAS

**Fig. 2:**
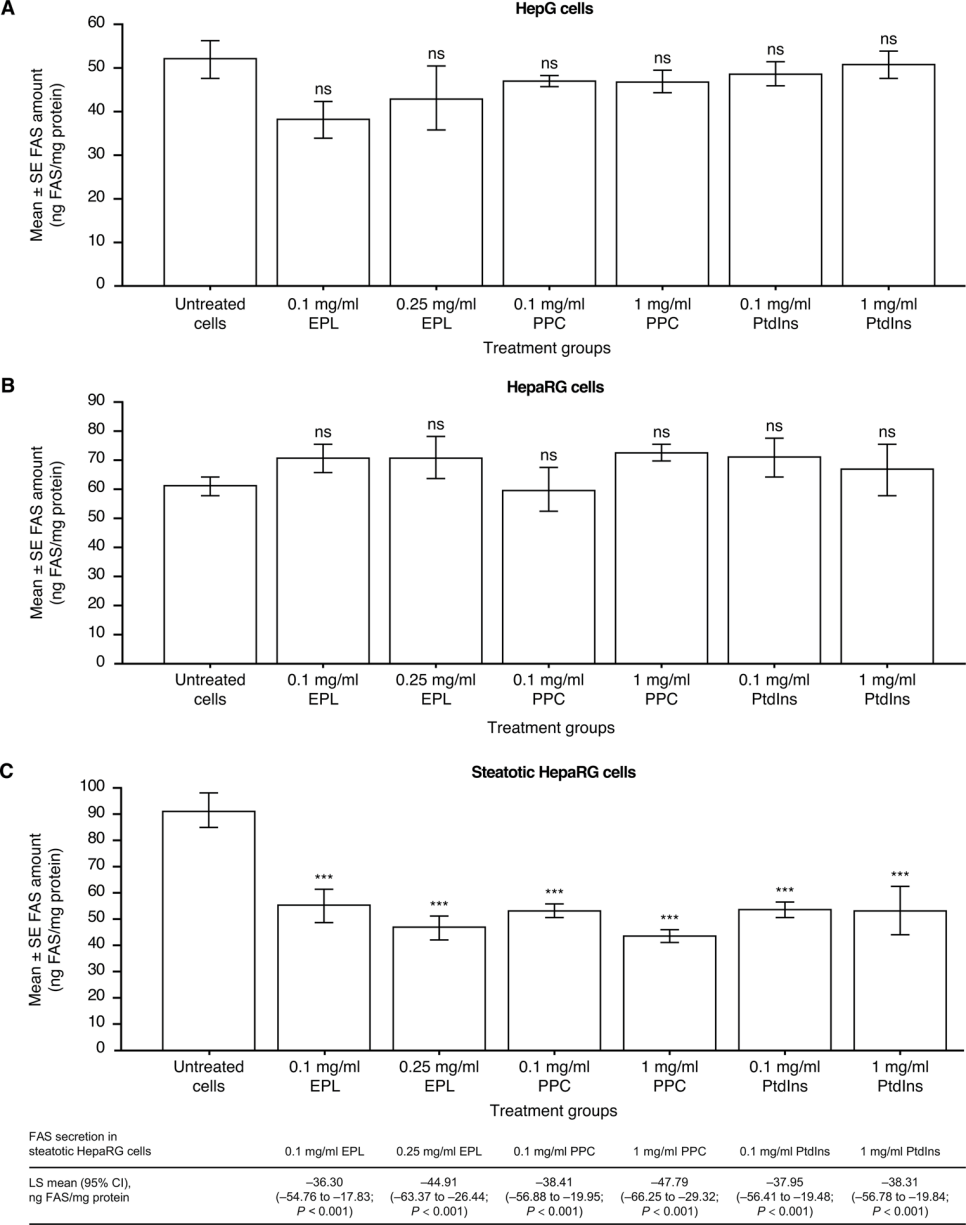
Effect of EPL, PPC, and PtdIns on the amount of FAS in (**A**) HepG2 cells; (**B**) HepaRG cells; and (**C**) steatotic HepaRG cells. Values shown are LS mean ± SE for four separate experiments; *n* = 1 well for each concentration of each compound per experiment. ***p < 0.001 versus untreated cells (ANOVA with Dunnett’s adjustment for pairwise comparisons). ANOVA, analysis of variance; EPL, essential phospholipid; LS, least squares; ns, not significant; PtdIns, phosphatidylinositol; PPC, polyenylphosphatidylcholine; SE, standard error

The amount of FAS in HepG2 cells was not impacted by phospholipid addition compared with untreated HepG2 cells (Fig. 2A).

In the absence of exogenous phospholipids addition, the FAS amount was significantly lower (LS mean difference [95% CI]) in HepaRG cells compared with steatotic HepaRG cells (–29.12 [–44.82, − 13.43] pg ACOX1/mg protein, *p* < 0.01). The amount of FAS in HepaRG cells was unaffected by exogenous phospholipids versus untreated HepaRG cells (Fig. 2B).

In steatotic HepaRG, all exogenous phospholipids at the concentrations assessed statistically significantly decreased the amount of FAS compared with untreated steatotic HepaRG (Fig. 2C).

### Impact of phospholipids on the amount of ACOX1

**Fig. 3:**
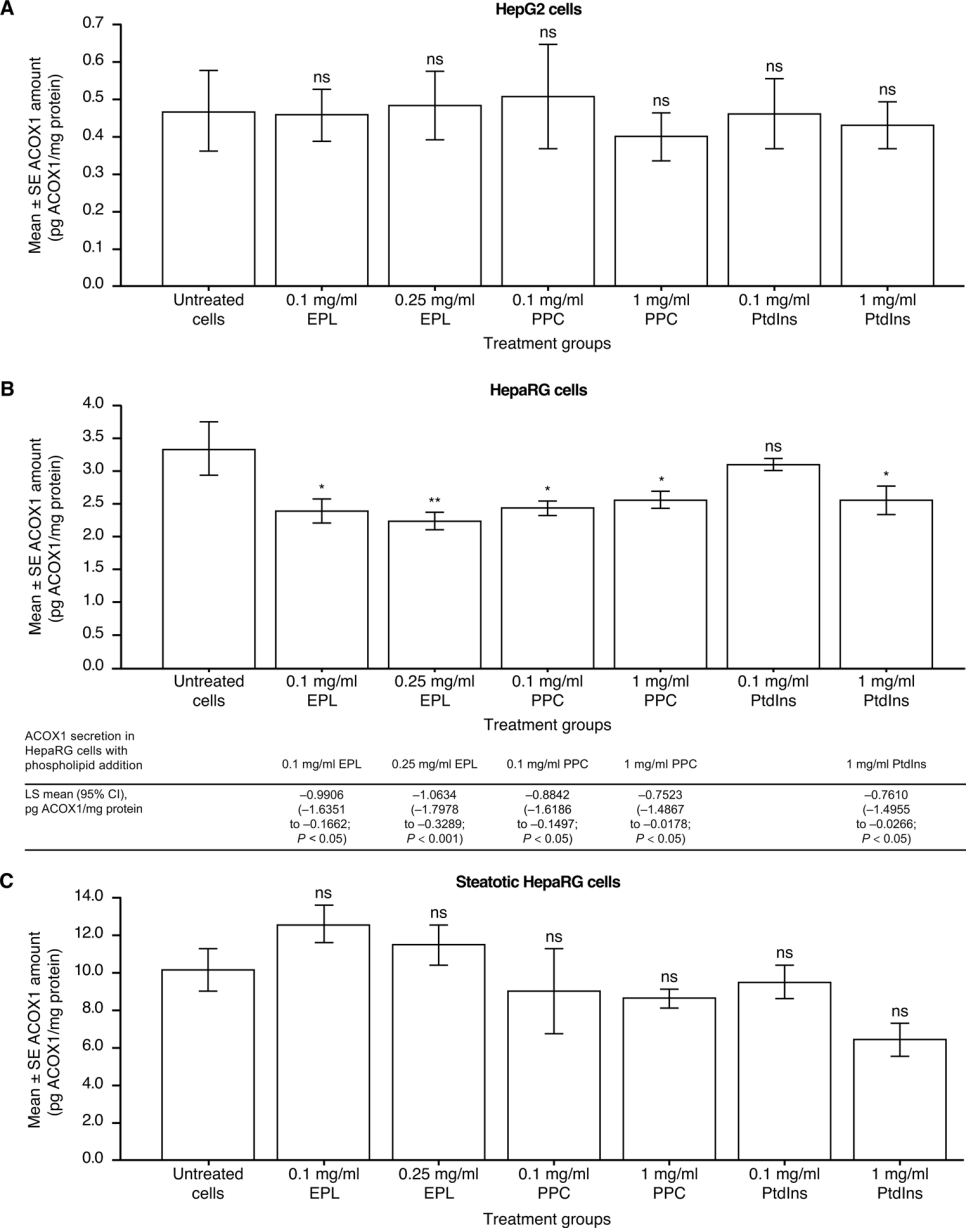
Effect of EPL, PPC, and PtdIns on the amount of ACOX1 in (**A**) HepG2 cells; (**B**) HepaRG cells; and (C) steatotic HepaRG cells. Values shown are LS mean ± SE for four separate experiments; *n* = 1 well for each concentration of each compound per experiment. *p < 0.05; **p < 0.01 versus untreated cells (ANOVA with Dunnett’s adjustment for pairwise comparisons). ACOX1, acyl-CoA oxidase; ANOVA, analysis of variance; EPL, essential phospholipid; LS, least squares; ns, not significant; PtdIns, phosphatidylinositol; PPC, polyenylphosphatidylcholine; SE, standard error

The amount of ACOX1 in HepG2 cells was unaffected by the addition of any of the phospholipids versus untreated HepG2 cells (Fig. 3A).

Without phospholipid addition, ACOX1 amount was significantly lower (LS mean difference [95% CI]) in HepaRG cells compared with steatotic HepaRG cells (–6.9136 [–9.1423, − 4.6849] pg ACOX1/mg protein, *p* < 0.001). With phospholipid addition to HepaRG cells, statistically significant decreases in the amount of ACOX1 were seen compared with controls, except for 0.1 mg/ml PtdIns (Fig. 3B).

The amount of ACOX1 in steatotic HepaRG cells was not affected by phospholipid addition versus controls (Fig. 3C).

### Impact of phospholipids on the amount of LCAT

**Fig. 4:**
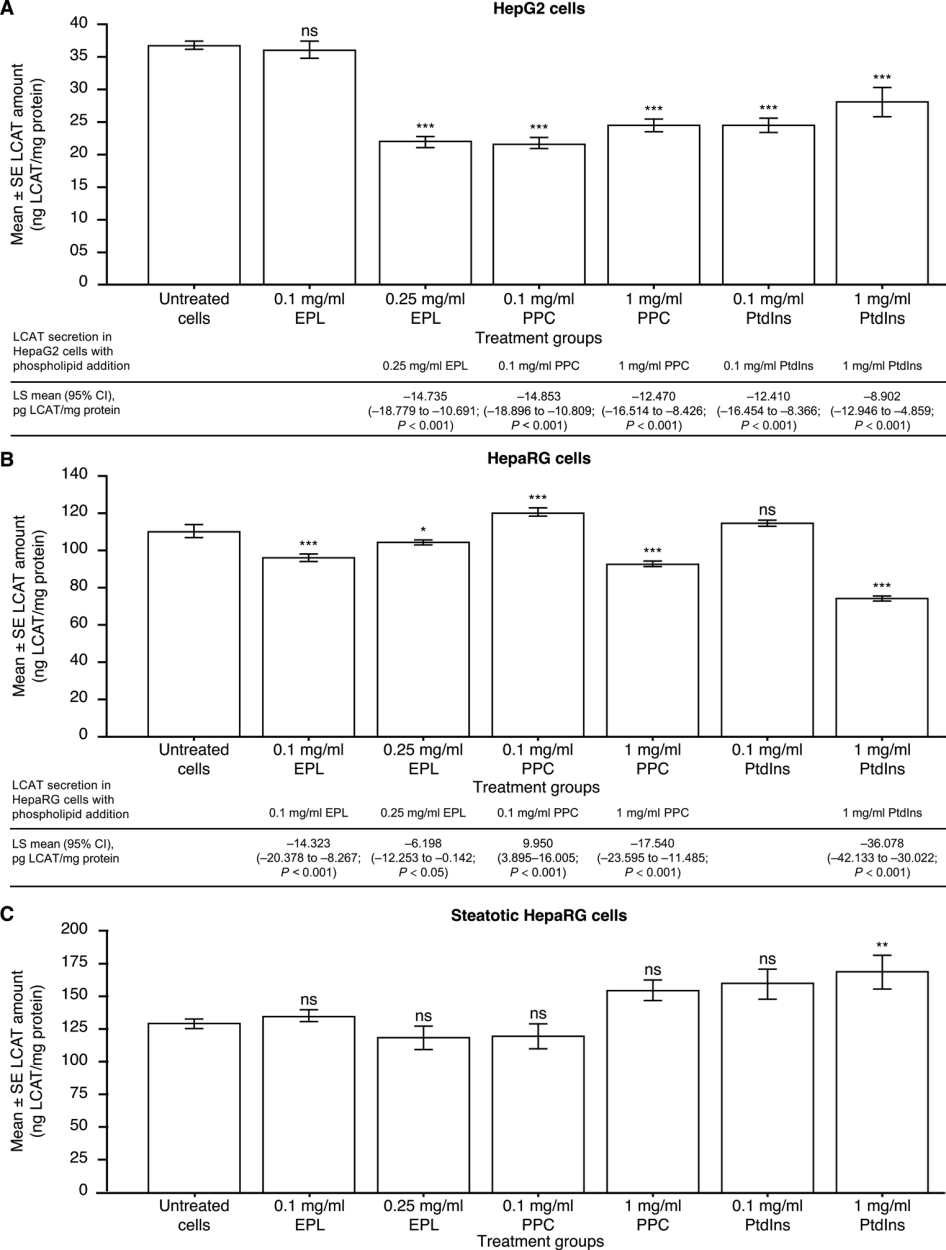
Effect of EPL, PPC and PtdIns on the amount of LCAT in (**A**) HepG2 cells; (**B**) HepaRG cells; and (**C**) steatotic HepaRG cells. Values shown are LS mean ± SE for four separate experiments; *n* = 1 well for each concentration of each compound per experiment. *p < 0.05; **p < 0.01; ***p < 0.001 versus untreated cells (ANOVA with Dunnett’s adjustment for pairwise comparisons). ANOVA, analysis of variance; EPL, essential phospholipid; LS, least squares; ns, not significant; PtdIns, phosphatidylinositol; PPC, polyenylphosphatidylcholine; SE, standard error

LCAT amount in HepG2 cells was statistically significantly decreased by all of the phospholipid additions, except for 0.1 mg/ml EPL, compared with untreated HepG2 cells (Fig. 4A).

Without phospholipid addition, LCAT amount was significantly lower (LS mean difference [95% CI]) in HepaRG cells compared with steatotic HepaRG cells (–17.952 [–27.614, − 8.291] ng LCAT/mg protein; *p* < 0.01). LCAT amount in HepaRG cells was statistically significantly changed by all of the phospholipid additions, except for 0.1 mg/ml PtdIns, compared with untreated HepaRG cells (Fig. 4B).

LCAT amount in steatotic HepaRG cells was not affected by the addition of either EPL or PPC compared with untreated cells (Fig. 4C). PtdIns addition to steatotic HepaRG cells had no effect on the amount of LCAT at 0.1 mg/ml, and statistically significantly increased the amount of this enzyme at 1 mg/ml: 39.487 (8.730, 70.245; *p* < 0.01) ng LCAT/mg protein versus untreated cells.

### Impact of phospholipids on G6PD activity

**Fig. 5:**
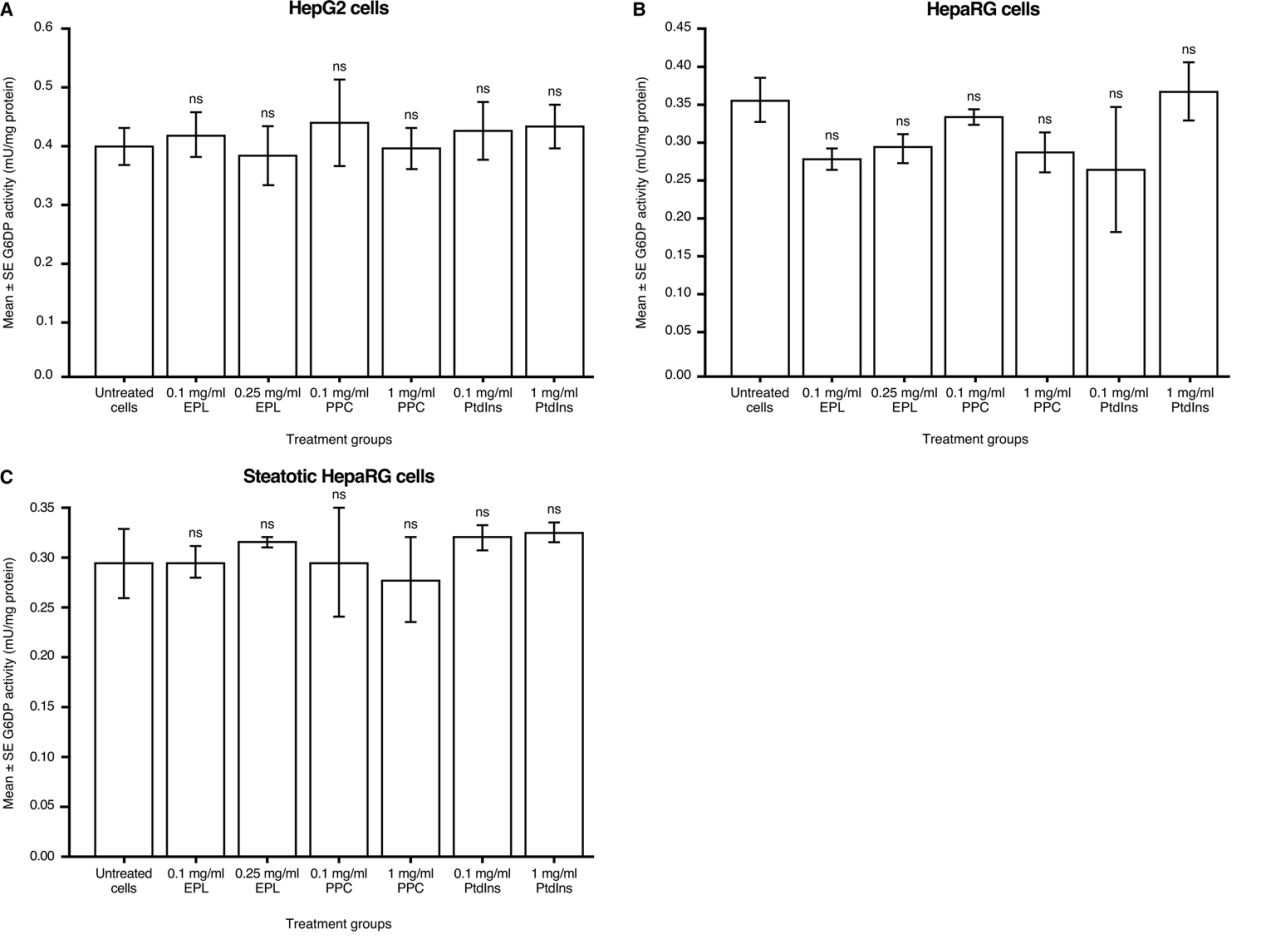
Effect of EPL, PPC and PtdIns on G6PD activity in (**A**) HepG2 cells; (**B**) HepaRG cells; and (**C**) steatotic HepaRG cells. Values shown are LS mean ± SE for four separate experiments; *n* = 1 well for each concentration of each compound per experiment. ANOVA with Dunnett’s adjustment for pairwise comparisons. ANOVA, analysis of variance; EPL, essential phospholipid; G6PD, glucose-6-phosphate dehydrogenase; LS, least squares; ns, not significant; PtdIns, phosphatidylinositol; PPC, polyenylphosphatidylcholine; SE, standard error

The addition of phospholipids, at any concentration evaluated, to HepG2 cells in culture had no significant effects on G6PD activity versus untreated HepG2 cells (Fig. 5A).

In the absence of phospholipid addition, G6PD activity (LS mean difference [95% CI]) was similar in HepaRG and steatotic HepaRG cells (0.07 [–0.03, 0.17] mU/mg protein). Adding phospholipids to HepaRG cells in culture had no significant effects on G6PD activity versus untreated cells (Fig. 5B).

For steatotic HepaRG cells in culture, phospholipid addition had no significant effects on G6PD activity versus untreated cells (Fig. 5C).

## Discussion

Using three human hepatocyte cell lines, the mechanism of action of EPL and two of its components on pro-inflammatory cytokines and lipid-metabolizing were investigated as inflammation and lipid dysregulation are part of NAFLD pathogenesis. LPS-induced IL-6 secretion was either significantly decreased (by all three phospholipids in HepaRG cells) or significantly increased (by 0.1 mg/ml EPL and 1 mg/ml PtdIns in steatotic HepaRG cells). All phospholipids tested significantly increased LPS-induced IL-8 secretion in both cell lines. FAS amounts were only significantly decreased by all phospholipids in steatotic HepaRG cells. ACOX1 amounts were unaffected in HepG2 and steatotic HepaRG cells and significantly decreased by all phospholipids in HepaRG cells. The amounts of LCAT were either significantly decreased (all phospholipids in HepG2 and HepaRG cells) or significantly increased (by 0.1 mg/ml PPC in HepRG cells and by 1 mg/ml PtdIns in steatotic HepaRG cells). Finally, G6PD activity was unaffected in all cell lines. These results expand previous investigations demonstrating that EPL, PPC, and PtdIns increased membrane fluidity, significantly reduced apoptosis, and increased hepatocellular extracellular transport involving certain transport proteins in human hepatocyte cell lines [22]. Collectively, these data provide insights into the EPL mechanism of action in improving liver function in various liver conditions, including NAFLD.

NAFLD is an inflammatory disease, particularly in the transition to NASH [46]. Several pro-inflammatory cytokines are involved in this disease progression [24, 25], including IL-6 [25, 26] and IL-8 [28, 47]. In the present investigations, only two pro-inflammatory cytokines (IL-6 and IL-8) could be reliably evaluated in two out of three human hepatocyte cell lines (HepaRG and steatotic HepaRG cells). Interestingly, secretion of IL-6 and Il-8 by steatotic HepaRG cells was significantly higher than by HepaRG cells under basal conditions, i.e., 3.4- and 1.5-fold higher, respectively. This finding is expected since steatotic HepaRG cells are an in-vitro model for hepatic steatosis [43] and provide support for the involvement of IL-6 and IL-8 in this process. Furthermore, these cell lines responded to the positive control LPS [18]. Indeed, LPS markedly enhanced IL-6 and IL-8 in both cell lines versus untreated cells (HepaRG: 418- and 97-fold higher, respectively; steatotic HepaRG: 30- and 28-fold higher, respectively). IL-8 secretion was considerably higher than IL-6 secretion by both cell lines under basal and LPS-stimulated conditions. In HepaRG cells, EPL, PPC, and PtdIns all significantly reduced LPS-stimulated IL-6 secretion. Contrastingly, LPS-stimulated IL-8 secretion was significantly increased by both concentrations of each phospholipid in HepaRG and steatotic HepaRG cells. Limited preclinical data are available for the impact of phospholipids on IL-6 and IL-8 secretion in animal models of liver inflammation. For example, in rats administered phosphatidylcholine (PtdCho; 600 mg/kg intraperitoneally) with and without LPS, LPS alone significantly increased serum pro-inflammatory cytokines (including IL-6) and induced significant histopathology changes in several organs (including the liver) [48]. PtdCho significantly reduced liver damage and serum IL-6 levels, as did hydrocortisone, a well-known anti-inflammatory agent [48]. In a mouse study, liver inflammation and fibrosis were reduced after PtdCho treatment, and IL-6 mRNA expression was decreased compared with controls [49]. The present results in HepaRG cells in which IL-6 secretion was significantly reduced by EPL, PPC, and PtdIns support the findings in these preclinical studies. In a study of 32 adults on long-term home parenteral nutrition regimens, SMOFlipid had no effect on liver function markers, blood lipids, or plasma cytokines, whereas ClinOleic significantly lowered gamma-glutamyltranspetidase and IL-8 concentrations [50]. Both lipid emulsion preparations altered the plasma profile of fatty acids [51]. Numerous investigations have shown EPL to have hepatoprotective benefits and that anti-inflammatory properties contribute to these benefits [7, 8, 11, 13, 14]. The present investigations directly demonstrate the anti-inflammatory effects of EPL and two of its components in a human hepatocyte cell line via reduction of IL-6 secretion. However, the reasons for the significant increase in LPS-induced IL-8 secretion (i.e., potentially pro-inflammatory) by all phospholipids tested in HepaRG and steatotic HepaRG cells are currently unknown. As IL-6 and IL-8 concentrations were measured in the same samples, the experimental conditions are an unlikely reason for this observation.

The liver is involved in lipid homeostasis [30], and lipid accumulation is a key part of NAFLD [29]. Furthermore, there is evidence that phospholipids (e.g., PtdCho) reduce steatosis via certain lipid metabolism pathways [51]. FAS is a key enzyme in fatty acid biosynthesis [31]. Importantly, dose-dependent lipid accumulation was achieved by incubating primary human hepatocytes in vitro with fatty acids [32]. Furthermore, FAS expression was also increased dose-dependently [32]. In the present in-vitro studies with human hepatic cell lines, FAS amounts were significantly higher in steatotic HepaRG cells versus HepaRG cells, which is in keeping with the findings from Dorn et al. [32]. In-vivo results showed that hepatic FAS expression was higher in mice with steatosis versus controls [32] and FAS activity was increased in rats with fatty liver [39]. A key finding in the present investigations in human hepatic cell lines is that the amount of FAS was significantly decreased in steatotic HepaRG cells following the addition of EPL, PPC, or PtdIns compared with untreated cells. This observation is in keeping with the finding that FAS activity in the fatty livers of rats was also decreased with PtdCho treatment compared with controls [39].

Changes in ACOX1, a key enzyme in peroxisomal fatty acid oxidation [33], have been linked to NAFLD in mice [34] and humans [35]. Evidence in a mouse model of alcoholic liver disease demonstrated that PtdCho administration significantly reduced ethanol-induced ACOX1 activity [16]. The present investigations showed that – whilst ACOX1 amounts were unaffected by EPL, PPC, or PtdIns addition to HepG2 and steatotic HepaRG cells – the amounts of this enzyme were significantly decreased by all of the phospholipids in HepaRG cells. Thus, there is now direct evidence of phospholipids impacting ACOX1 in isolated human hepatocytes as part of the mechanism of EPL and its components in improving liver function.

Regarding LCAT, several investigations have evaluated the impact of phospholipids on this enzyme. Oral administration of polyunsaturated lecithin apparently activated LCAT in chimpanzees [52]. Synthesized species of PtdCho with differing fluidity were used to form recombinant high-density lipoproteins and the impact on human LCAT activity was assessed [53]. This evaluation showed that decreasing fluidity correlated with decreased LCAT activity; thus, PtdCho fluidity and structure may be a key regulator of this enzyme [53]. Rats fed diets containing phospholipids demonstrated significant increases in activity of LCAT in the plasma [54–56]. LCAT activity was inhibited by PtdIns compared with controls, whereas PtdCho had no effect in rabbits injected with these phospholipids [57]. In the present evaluations, LCAT amounts were significantly decreased by EPL, PPC, and PtdIns in HepG2 and HepaRG cells, whilst 0.1 mg/ml PPC (HepRG cells) and 1 mg/ml PtdIns (steatotic HepaRG cells) significantly increased the amount of LCAT. These in-vitro observations do not match those of previous findings reporting increased LCAT activity [53, 56]. The reasons for this discrepancy are unknown, although the amount of LCAT in the human cell lines was measured rather than LCAT activity per se.

The final enzyme evaluated in human hepatocyte cell lines was G6PD activity. EPL, PPC, and PtdIns had no impact on G6PD activity in any of the human hepatocyte cell lines. Moreover, G6PD activity was similar between the HepaRG and steatotic HepaRG cells. These observations are in contrast to an in vivo study in rats [39]. In rats fed a diet supplemented with orotic acid for 10 days, fatty liver was induced and one of several other changes was a significant increase in hepatic G6PD activity compared with rats fed a regular diet [39]. Importantly, in rats receiving PtdCho (as 20% of dietary lipid) and orotic acid in the diet, there was no lipid accumulation in the liver and hepatic G6PD activity was similar to controls [39]. It is unknown why EPL and its components had no effect on G6PD activity in human hepatocyte cell lines.

There were clear differences between the three human hepatocyte cell lines on the impact of EPL, PPC, and PtdIns. Such differences may reflect the fact that differentiated HepaRG cells have different levels of expression of drug-metabolizing enzymes and drug transporters versus HepG2 cells [58]. The findings on the effects of phospholipids on pro-inflammatory cytokines and lipid-metabolizing enzymes across the cell lines used should therefore be considered to obtain an overview of the effects of PLs on liver function in vitro. Some of the current findings with phospholipids did not correlate with previously reported results from in-vivo studies. Such differences may be explained by species differences, or that some effects may only be seen in vivo given the complexity and interactions between biochemical pathways which cannot be reproduced in vitro. Another explanation resides in potential differences in phospholipid concentrations at the target site in vivo versus those used in vitro. Nevertheless, the current findings in human hepatocyte cell lines are valuable in evaluating the mechanism of action of EPL and two of its components in improving liver function in NAFLD.

These investigations have several strengths. In-vitro techniques are of great value in elucidating the mechanism of action of the compound(s) of interest. The present in-vitro evaluations, and those reported by Wupperfeld et al. [22], provide the first direct evidence of the impact of EPL and some of its components on human hepatocytes, thereby providing insight into the clinical effects of EPL in patients with NAFLD. Several human hepatocyte cell lines were utilized, allowing for data extrapolation to patients with NAFLD. However, based on gene expression in HepG2, HepaRG and primary human hepatocytes, HepaRG cell lines more closely resemble normal liver [41, 59, 60]. As with all investigations, there are certain weaknesses in the current evaluations. Immortal cell lines, derived from human liver cancer, were used which are unlikely to fully represent the function of the normal liver. Indeed, it is known that HepG2 and HepaRG cells have differences in drug-metabolizing enzymes, drug transporters, and gene expression profiles [58, 60]. However, it is not feasible to use primary cultures of human hepatocytes. The concentrations of EPL, PPC, and PtdIns in hepatocytes in vivo following oral administration are unknown; thus, it is not possible to extrapolate the concentrations used to in vivo concentrations. However, the maximum, non-cytotoxic concentrations of each phospholipid were used in the current evaluations.

In conclusion, the current investigations highlighted changes in pro-inflammatory cytokines and lipid-metabolizing enzymes in response to EPL and PPC and PtdIns in human hepatocyte cell lines in vitro. This includes anti-inflammatory changes and anti-steatotic changes in the specific cell lines. These data add to previous findings that these phospholipids increased membrane fluidity, decreased apoptosis, and increased function of hepatocellular extracellular transporters in these cell lines [22]. Such changes in these endpoints may help improve liver function, and provide further insights into the mechanism of action of EPL as a hepatoprotective, adjuvant treatment for NAFLD.

### Electronic supplementary material

Below is the link to the electronic supplementary material.

Supplementary Material 1

## Data Availability

Qualified researchers may request access to data and related study documents including the study report, study protocol with any amendments, statistical analysis plan, and dataset specifications. Further details on Sanofi’s data sharing criteria, eligible studies, and process for requesting access can be found at: https://www.vivli.org/.

## Abbreviations

ACOX1: Acyl-CoA oxidase
ANOVA: Analysis of variance
CI: Confidence interval
EPL: Essential phospholipids
G6PD: Glucose-6-phosphate dehydrogenase
IL: Interleukin
LPS: Lipopolysaccharide LS: Least-square
NAFLD: Non-alcoholic fatty liver disease
NASH: Non-alcoholic steatohepatitis
PPC: Polyenylphosphatidylcholine
PtdCho: Phosphatidylcholine
PtdIns: Phosphatidylinositol
