# Supplementary material for: Essential phospholipids impact cytokine secretion and alter lipid-metabolizing enzymes in human hepatocyte cell lines
Source: Pharmacol Rep. 2024 Apr 26;76(3):572–84. doi: 10.1007/s43440-024-00595-4 (PMC11126482; doi:10.1007/s43440-024-00595-4)
Supplement: Supplementary file 1 — Supplementary Material 1 [file 43440_2024_595_MOESM1_ESM.docx]

**Fig. S1** Dose-response and time course of LPS induction of IL-8 secretion in HepaRG cells


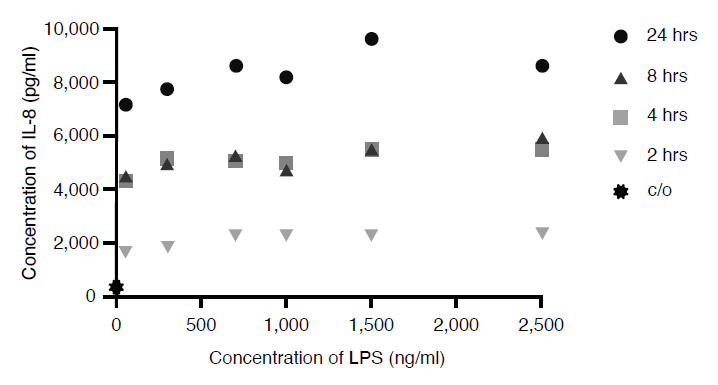


Experiment was not carried out as quadruplicate.

c/o, cell only i.e., untreated cells (control); IL, interleukin, LPS, lipopolysaccharides.

**Supplementary Information**

**Chemicals and reagents**

DMSO, insulin, stearic acid, and oleic acid were obtained from Sigma-Aldrich (St Louis, MO, USA). Hydrocortisone hemisuccinate was purchased from Santa Cruz Biotechnology (Dallas, TX, USA). Lipopolysaccharide (LPS) from *Escherichia coli* O111:B4 was provided by Sigma-Aldrich. Penicillin/streptomycin, FBS were purchased from Biochrom (Berlin, Germany). EPL (Essentiale Forte 300 mg) was obtained from Sanofi. PPC (LIPOID S 80, phospholipids from soybeans with 75% polyphosphatidylcholine) and PtdIns (ALCOLEC^®^ H PI 35 P, fat-free sunflower lecithin [non‑genetically modified organism] with ~35 % phosphatidylinositol) were obtained from Lipoid (Ludwigshafen am Rhein, Germany). All other chemicals were purchased from commercial sources and were of the highest purity available.

The mean (standard deviation, mol/%) of the components for Essentiale are: 61.94 (2.23) phosphatidylcholine; 16.18 (1.33) lysophosphatidylcholine; 4.85 (0.48) phosphatidylethanolamine; 0.47 (0.22) PtdIns; 0.31 (0.04) phosphatidylglcerol; 0.40 (0.06) phosphatidic acid; 1.31 (0.38) diacylglycerol; and 13.50 (2.40) triacylglycerol (10).

**Preparation of liposomes**

In brief, dual centrifugation (ZentriMix 380 R, Andreas Hettich GmbH &Co KG, Tuttlingen, Germany) was used to prepare liposomes as described by Massing *et al.* [1]. SiLibeads (TypZY-P 1.4–1.6 mm, Art.No.:
9715-41, Sigmund Lindner GmbH, Warmensteinach, Germany) were added at 1000% (or a minimum of 1 g) versus the phospholipid mass (set at 100%), and cell culture medium was added at 150% of the phospholipid mass. This mixture was dual centrifuged (15 min, 2340 rpm). Additional centrifugations (3 min, 2340 rpm) were conducted with cell culture medium volumes of 300% (second run) and 550% (third run) of phospholipid mass. The resulting dispersions of liposomes were diluted to 50 mg/ml phospholipid concentration and filtered using a mixed cellulose esters member (pore size 0.45 µm; Merck KGaA, Darmstadt, Germany).

**Cell lines and culture conditions**

- HepG2 cells: Sigma-Aldrich (Acc No: 85011430, Lot: 16K046; St. Louis, MO, USA)
- Roswell Park Memorial Institute 1640 medium: Thermo Fisher Scientific GmbH, Waltham, MA, USA
- Fully differentiated HepaRG cells: Lonza; Cat. No: NSHPRG, Walkersville, MD, USA
- Williams’ medium E: Merck KGaA, Darmstadt, Germany

**ELISA kits**

Human FAS ELISA kits (ab279412) were purchased from abcam. Human ACOX1 ELISA kits (abx585105) was purchased from Abbexa Ltd (Cambridge, UK). Both of these methods are based on the sandwich ELISA technology and the procedure was very similar to the LCAT method described below. These assays were conducted as per the manufacturer’s instructions.

Human LCAT ELISA kits (CSB-E13469h) were purchased from Cusabio (Houston, TX, USA). This assay is a quantitative sandwich enzyme immunoassay and was performed according to the manufacturer’s instructions. Briefly, standards and samples were added to the wells coated with antibody, which binds any LCAT present. Unbound substances were then removed by washing, and a biotin-conjugated antibody specific for LCAT was added to the wells. After washing, avidin-conjugated horseradish peroxidase was added to the wells. Following a wash to remove any unbound avidin-enzyme reagent, a tetra methyl benzidine substrate solution was added to the wells. The developed color is proportional to the amount of LCAT bound in the initial step. Color development was stopped by adding the acidic stop solution and the intensity of the color was measured spectrophotometrically at 450 nm using a Tecan Sunrise Vis-photometry plate reader (Tecan, Männedorf, Switzerland).

G6PD activity assay kits (ab176722) was purchased from abcam (Cambridge, UK) and the assay was performed according to the manufacturer’s instructions. In this assay, G6PD present in the sample converts NADP+ to NADPH, which is monitored by a fluorogenic NADPH sensor to yield a highly red fluorescence product. The signal was detected at excitation/emission of 540/590 nm in a fluorescent microplate reader (Tecan Group Ltd, Männedorf, Switzerland) The fluorescence intensity is proportional to G6PD activity.

FAS, ACOX1 and LCAT were reported as the amount of enzyme per unit of cellular protein based on the assumption that all cells have the same amount of protein. Pierce^TM^ BCA protein assay kits were obtained from Thermo Scientific^TM^ (Waltham, MA, USA), which quantifies protein colorimetrically using bicinchoninic acid. The resulting purple-colored reaction product exhibits strong absorption at a wavelength of 562 nm. Protein standard (bovine serum albumin) solutions (25–2000 µg/ml), and test samples were added to a 96-well plate. The working reagent was then added to each well, and the plate was then carefully shaken and incubated at 37°C for 30 min. The absorbance was detected in the Tecan Sunrise Vis-photometric plate reader (Tecan, Männedorf, Switzerland) at a wavelength of 562 nm.

**Additional equipment**

- T-75 flasks: Greiner Bio-One GmbH, Frickenhausen, Germany
- 12-well culture plates: Corning, Kaiserslautern, Germany
- 6-well culture plates: Corning, Kaiserslautern, Germany

**Reference**

1. Massing U, Ingebrigtsen SG, Škalko-Basnet N, Holsæter AM. Dual centrifugation – a novel ‘in-vial’ liposome processing technique. In: Catala A, editor. Liposomes. IntechOpen, London, UK; 2017. p. 3–28.
